# Serum angiotensin type-1 receptor autoantibodies and neurofilament light chain as markers of neuroaxonal damage in post-COVID patients

**DOI:** 10.3389/fimmu.2025.1571027

**Published:** 2025-04-22

**Authors:** Ana I. Rodriguez-Perez, Gemma Serrano-Heras, Carmen M. Labandeira, Laura Camacho-Meño, Beatriz Castro-Robles, Juan A. Suarez-Quintanilla, Mónica Muñoz-López, Pepa Piqueras-Landete, María J. Guerra, Tomas Segura, José L. Labandeira-Garcia

**Affiliations:** ^1^ Research Center for Molecular Medicine and Chronic Diseases (CIMUS), University of Santiago de Compostela, Santiago de Compostela, Spain; ^2^ Research Health Institute of Santiago (IDIS), Santiago de Compostela, Spain; ^3^ Networking Research Center on Neurodegenerative Diseases (CIBERNED), Madrid, Spain; ^4^ Research Unit, General University Hospital of Albacete, Albacete, Spain; ^5^ Neurology Service, University Hospital of Ourense, Ourense, Spain; ^6^ Primary Health-Care Unit Fontiñas, IDIS, University of Santiago de Compostela, Santiago de Compostela, La Coruña, Spain; ^7^ Faculty of Medicine, University of Castilla-La Mancha (UCLM), Albacete, Spain; ^8^ Biomedicine Institute (IB-UCLM), Albacete, Spain; ^9^ Department of Neurology, General University Hospital of Albacete, Albacete, Spain

**Keywords:** autoantibody, autoimmunity, AT1, biomarkers, cognitive impairment, COVID-19, neurological long-COVID, post-acute sequelae of COVID-19 syndrome

## Abstract

**Introduction:**

Dysregulation of autoimmune responses and the presence of autoantibodies (AA), particularly those related to the renin-angiotensin system (RAS), have been implicated in the acute phase of COVID-19, and persistent dysregulation of brain RAS by RAS-related autoantibodies may also contribute to neurological symptoms of post-COVID.

**Methods:**

We analyzed levels of serum and CSF RAS AA in post-COVID patients with neurological symptoms, individuals who have fully recovered from COVID-19 (after-COVID controls), and uninfected individuals, and their possible correlations with the serum marker of neuroaxonal damage neurofilament light chain (NfL) and the degrees of cognitive deficit.

**Results:**

Both in serum and CSF, levels of AA agonists of the pro-inflammatory angiotensin II type 1 receptors (AT1-AA) were significantly elevated in this cohort of neurological post-COVID patients compared to both uninfected and after-COVID controls and correlated with serum levels of NfL. Changes in serum and CSF levels of AA promoting the RAS anti-inflammatory axis (upregulation of AA agonists of AT2 and Mas receptors, downregulation of AA antagonists of ACE2) suggest upregulation of the RAS compensatory response in this cohort of neurological post-COVID patients. Post-COVID patients with more pronounced cognitive impairment exhibited significantly higher CSF levels of MasR-AA and a trend toward elevated AT2-AA. Persistent brain RAS dysregulation, particularly persistent increase in AT1-AA, and its correlation with neuroaxonal damage markers and cognitive impairment, may play a significant role in neurological symptoms associated with post-COVID. Serum levels of NfL and AT1-AA may be interesting biomarkers for the early identification of CNS involvement in patients with neurological symptoms and a history of COVID-19. However, post-COVID is a highly heterogeneous entity and may result from various underlying mechanisms. The present study includes a cohort, which may differ from other cohorts with different clinical profiles, which may show different results on NfLs and CSF RAS autoantibodies, particularly AT1-AA.

**Conclusion:**

These findings highlight the potential of targeting AT1 receptors as a therapeutic strategy for mitigating cognitive deficits in post-COVID patients showing upregulated AT1-AA levels.

## 1 Introduction

A considerable proportion of patients who had COVID-19 develop post-infectious sequelae, which have been referred to by various names (1). According to a broad consensus and in alignment with the nomenclature specified in the present research topic, in this study, we refer to post-COVID patients as those experiencing symptoms persisting from three months to several years after COVID-19, and long-COVID patients as those with symptoms lasting only two to three months after the infection. Although long-COVID and post-COVID are highly heterogeneous entities, they commonly manifest with a range of neurological symptoms, including cognitive impairment, chronic fatigue, and neuropsychiatric conditions such as depression and anxiety. A frequent feature is persistent cognitive impairment, colloquially called “brain fog” (2). However, the underlying mechanisms responsible for these neurological manifestations, particularly cognitive deficits, remain poorly understood. Several potential etiologies have been proposed, including residual effects from the acute phase of the disease, such as microvascular injury or hypoxia (3), as well as persistent neuroinflammation and the sustained release of inflammatory cytokines (4). These processes may lead to damage in both gray and white matter, as well as disruption of adult hippocampal neurogenesis. Consistent with this, recent studies have reported a reduction in gray or white matter in several brain regions associated with cognitive dysfunction, including hippocampal volume loss (5, 6), although some studies have yielded conflicting results (7).

**Figure 1:**
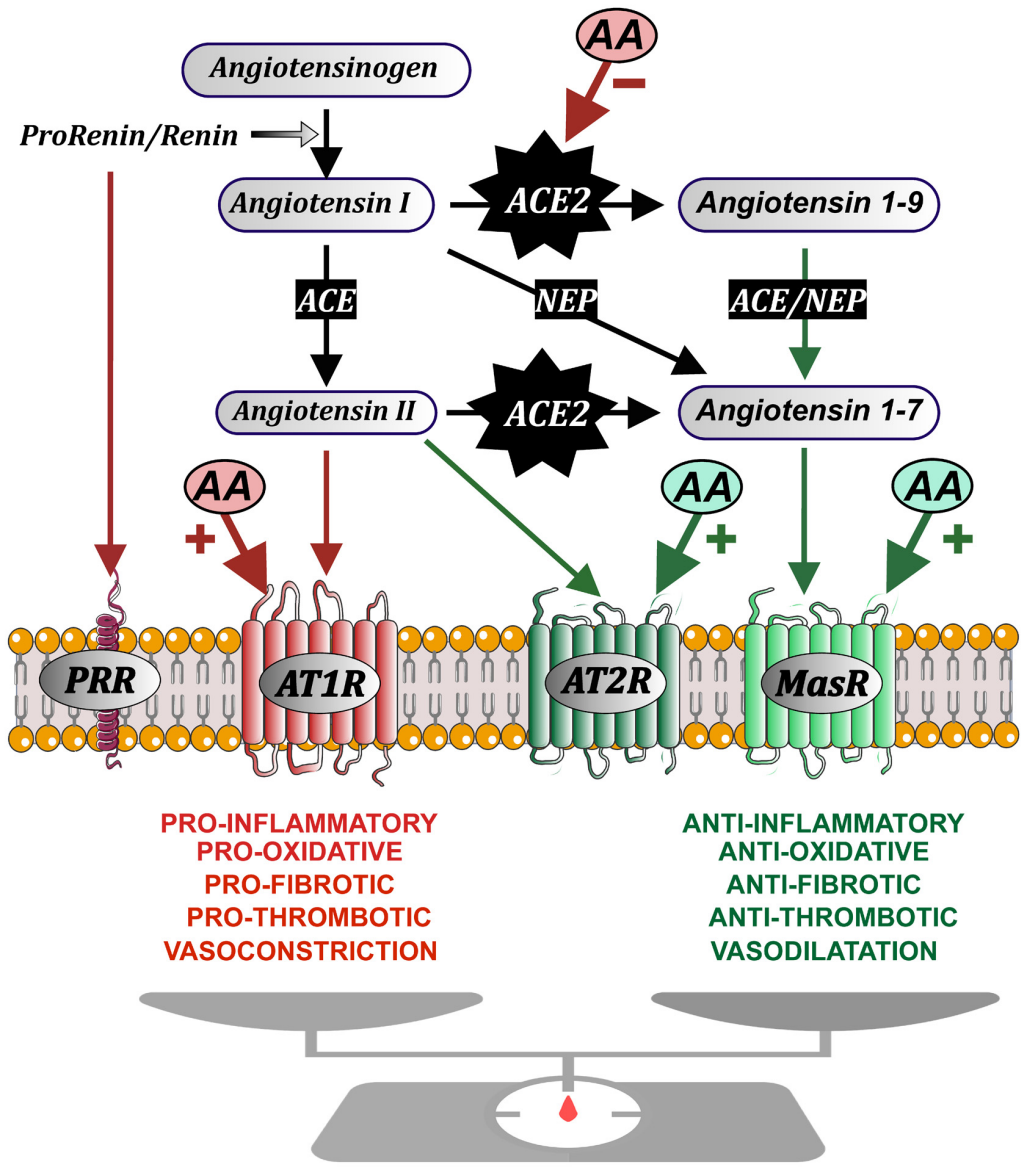
Schematic organization of the renin-angiotensin system (RAS), which is formed by two opposite axes: a first axis (red arrows) mainly constituted by Angiotensin II acting on AT1 receptors (AT1R), and a compensatory axis (green arrows) constituted by Angiotensin II acting on AT2 receptors, and Angiotensin 1-7 acting on Mas receptors. Angiotensin II is produced by the enzyme prorenin/renin acting on the precursor protein angiotensinogen to produce Angiotensin I, which is converted by the angiotensin-converting enzyme (ACE) into Angiotensin II. Renin and its precursor prorenin (PR) can also act on specific PR receptors. Angiotensin-converting enzyme 2 (ACE2) plays a key role in the system balance because ACE2 (together with peptidases such as Neprilysin, NEP) transforms components of the pro-inflammatory axis (Angiotensin I and, particularly, Angiotensin II) into components of the compensatory axis (Angiotensin 1-9 and, particularly Angiotensin 1-7). Autoantibodies (AA) play an agonistic effect on the GPCR (G protein-coupled receptors: AT1, AT2, Mas) and antagonistic effect on ACE2. Figure modified with permission from: Drugs Modulating Renin-Angiotensin System in COVID-19 Treatment. J.L. Labandeira-Garcia et al., 2022, Biomedicines, 10 (DOI:10.3390/biomedicines10020502).

Dysregulation of autoimmune responses and the presence of autoantibodies (AA), particularly those related to the renin-angiotensin system (RAS), have been implicated in the acute phase of COVID-19 (8, 9), and they may also play a role in the post-COVID syndrome (10, 11). The RAS has been identified as a critical player in the pathogenesis of COVID-19 (12, 13). A central component of the RAS, angiotensin-converting enzyme 2 (ACE2), serves as the entry receptor for SARS-CoV-2 (14), and its interaction with the virus leads to RAS dysregulation, favoring the pro-inflammatory arm of the system and promoting organ inflammation. The tissue RAS, including the brain RAS, consists of two opposing axes: a first axis, primarily driven by angiotensin II (AngII) acting on angiotensin type 1 (AT1) receptors, promotes inflammatory, oxidative, fibrotic, thrombotic, and vasoconstrictive effects. A second or compensatory axis, which includes AngII acting on AT2 receptors and angiotensin 1-7 (Ang1-7) acting on Mas receptors (MasR), counteracts the effects of the AT1 activity (15, 16). ACE2 converts pro-inflammatory peptides such as AngII into anti-inflammatory peptides such as Ang1-7, and viral binding to ACE2 causes its downregulation, shifting the RAS balance towards the pro-inflammatory axis (17). The general organization of tissue RAS is summarized in **Figure 1**.

In the context of COVID-19, our studies (9, 12), as well as those from other groups, have shown elevated levels of autoantibodies targeting RAS GPCR (G protein-coupled receptors: AT1, AT2, MasR), and autoantibodies acting on ACE2, which were associated with severe COVID-19 outcomes (18–20). Several previous studies have shown RAS GPCR autoantibodies act as agonists on the corresponding receptors, while ACE2 autoantibodies have antagonistic effects on this enzyme activity (19, 21–24). The strong agonistic effect of AT1-AA was initially demonstrated in preeclampsia and preeclampsia models (23, 25) and later in other processes (26, 27). In several recent studies using cell cultures and animal models, we showed that AT1-AA, isolated and purified from serum of preeclamptic women, enhanced the pro-oxidative and proinflammatory effects of AT1 in neurons and glial cells leading to an increase in neuron degeneration and neuroinflammation as well as blood-brain barrier (BBB) disruption (28–30).

Persistent dysregulation of the brain RAS by RAS-related autoantibodies, after the acute phase of COVID-19, may contribute to the neurological symptoms of post-COVID. The present study aims to analyze the potential role of autoantibodies targeting RAS in the pathophysiology of the post-COVID neurological syndrome by comparing the levels of RAS autoantibodies in the serum and cerebrospinal fluid (CSF) of post-COVID patients with neurological symptoms, individuals who have fully recovered from COVID-19 (after-COVID controls), and uninfected individuals. We further explored possible correlations between RAS autoantibodies and the serum marker of neuroaxonal damage neurofilament light chain (NfL), and the degrees of cognitive deficit in a cohort of patients with neurological post-COVID.

## 2 Materials and methods

### 2.1 Study design and participants

This is a cross-sectional study with clinical cases, patients, and two groups of controls, involving two centers: the General University Hospital of Albacete and the Primary Health-Care Unit Fontiñas (Santiago de Compostela, Spain). Patients who suffered from mild to moderate COVID-19 disease (evidence as PCR or antigen positive) during the initial waves (2020 and the first trimester of 2021) and had long-term neurological symptoms for more than 9 months before inclusion were consecutively recruited through the Department of Neurology at the Hospital of Albacete and identified in the present study as “post-COVID group”. A second group, identified in this study as “after-COVID controls”, included individuals with a history of SARS-CoV-2 infection with symptoms ranging from mild to severe, who did not experience any persistent symptoms before their inclusion in the study. This research also included a third group with no history of SARS-CoV-2 infection who did not exhibit neurological manifestations (“uninfected controls”). These groups were enrolled at the Hospital of Albacete and the Primary Health-Care Unit Fontiñas (Santiago de Compostela, Spain). Exclusion criteria for all participants included: (a) a diagnosis of another neurological, psychiatric, or systemic illness that could explain the neurological symptoms; (b) a previous diagnosis of chronic fatigue syndrome or fibromyalgia; (c) immunosuppression and/or corticosteroid treatment before the blood or CSF test; and (d) an educational level below primary school. All participants provided informed consent and agreed to undergo blood and cerebrospinal fluid (CSF) collection, while patients with post-COVID were also subjected to a clinical evaluation and neurocognitive assessment. Ethics: This study was conducted following the Declaration of Helsinki for human research, the EU Regulation 2016/679, and the Spanish Organic Law 3/2018 on the protection of personal data and received approval from the local Medical Research Ethics Committee (Acta 02/2021). The privacy rights of all participants in the study were strictly observed.

### 2.2 Clinical and cognitive examinations

The evaluation was conducted by a neurologist and two trained neuropsychologists and began with a clinical interview covering the patient’s medical history, symptoms experienced during the acute phase of COVID-19, the frequency of these symptoms (recorded using a questionnaire detailing 27 common post-COVID manifestations), functional status, and demographic information. Subsequently, patients with post-COVID underwent cognitive screening using the Addenbrooke’s Cognitive Examination (ACE III) (31, 32), which assesses the domains of attention, verbal fluency, visuospatial processing, language, learning, and memory, ultimately providing an Overall Cognitive Level (OCL) score. Patients were then classified into three cognitive profile categories according to the guidelines of the American Academy of Clinical Neuropsychology (33) as follows: 1) Cognitively unimpaired: average score, z > -0.71, equivalent to the >24th percentile (pc); 2) Mildly impaired: low average score, z ≤ -0.71, equivalent to the ≤24th percentile; and 3) Severely impaired: below average score, z ≤ -1.40, equivalent to the ≤8th percentile.

### 2.3 Collection of serum and CSF samples and molecular analysis

Blood samples were obtained by venipuncture and collected in Vacutainer Tube SST II Advance Serum Separator Gel 8.5 mL (Ref. 366468). The samples were centrifuged at 1,500 G for 7 minutes to isolate serum. CSF samples were obtained by expert neurologists through lumbar puncture under aseptic conditions, collected in polypropylene tubes, and then centrifuged at 400 g for 10 minutes to remove cellular debris and aggregates. The resulting serum and CSF samples were immediately frozen and stored at −80°C until further analysis. For this study, we obtained 79 serum samples and 21 CSF samples from uninfected controls, 24 serum samples and 7 CSF samples from after-COVID controls; and 69 serum and 69 CSF samples from all patients with post-COVID.

Serum and CSF levels of AT1-AA, ACE2-AA, AT2-AA, and MasR-AA were measured using commercial-specific solid-phase, sandwich enzyme-linked immunosorbent assays (ELISA) from CellTrend; Luckenwalde, Germany. The batch effects were minimized by balancing the groups between plates. The manufacturer’s instructions were strictly followed. Absorbance was measured at 450/620 nm using an Infinite M200 multiwell plate reader (TECAN, Männedorf, Switzerland), and autoantibody concentrations were quantified as arbitrary units (U) by extrapolating from a standard curve composed of five to seven standards (ranging from 0.625 to 100 U/mL, depending on the autoantibody) using a 4PL curve fit. No threshold is provided by the manufacturer, who recommends that each laboratory establish its reference range for the tested population. In all cases, samples with values over the standard curve were diluted with assay buffer to get their absorbances within the standard curve. The ELISA kits were validated according to the Food and Drug Administration’s Guidance for Industry: Bioanalytical Method Validation, and in many previous studies.

Serum Neurofilament light chain (NfL) levels were quantified using the ultra-sensitive Single Molecule Array (SIMOA) technology on the Simoa SR-X platform (Quanterix Corp, Billerica, Massachusetts, USA). The human NF-light assay kit (Cat No: 104364) was used according to the manufacturer’s protocol. All samples were analyzed on the same day using the same batch of reagents. A four-parameter logistic curve-fitting data reduction method was employed to generate a calibration curve. Two control samples with known concentrations of NfL (high-control and low-control) were included for quality control purposes. The operators were blinded to the participants’ disease status and clinical information.

### 2.4 Statistical analysis

Univariate data analysis was performed using SigmaPlot 11.0 (Systat Software, Inc., CA, USA), while multivariate regression models were performed with R Software Version 4.4.2. Scatter dot plot graphs were generated using GraphPad Prism 8 (GraphPad, Inc., San Diego, CA, USA). The sample size was calculated considering a standard effect size (34). Accepting an alpha risk of 0.05 and a power of 0.8 in a two-tailed test, 25 subjects are necessary in each group to recognize as statistically significant a large difference (f = 0.4) on NfL levels between any pair of the 3 groups considered. A 15% correction factor has been applied as this variable is assumed not to be normally distributed, and a non-parametric test must be used for comparisons. The median and interquartile range (IQR) were used as central tendency and dispersion estimators, respectively. The normality of the data was assessed using the Kolmogorov-Smirnov test. For multiple comparisons, data did not meet the assumptions of normality and homogeneity of variances, so they were analyzed using the Kruskal–Wallis one-way ANOVA on ranks followed by Dunn´s *post-hoc* test. However, pairwise comparisons were performed using a two-sample t-test for continuous variables or a Chi-square test for categorical variables, as appropriate. Spearman’s coefficients were used to study correlations between different parameters. When comparing groups, multiple linear regression analyses were conducted to account for potential confounding factors. The response variable was log-transformed when necessary to help the models meet the assumptions of normality and homoscedasticity of residuals. These assumptions were checked for each model to ensure validity. P-values less than 0.05 were considered statistically significant.

## 3 Results

### 3.1 Demographic and clinical and characteristics of the study cohort

This study included 99 individuals who had no record of SARS-CoV-2 infection and did not show any neurological signs (uninfected controls), 28 COVID-19-recovered individuals not suffering from persistent neurological symptoms (after-COVID controls) and 69 patients with neurological post-COVID. Demographic variables are shown in **Supplementary Table S1**. The mean age of the uninfected control group was 61.3 ± 9.2 years, while the after-COVID controls and post-COVID patients had mean ages of 53.4 ± 14 and 52.5 ± 8.1 years, respectively. Females represented 51.5% of the uninfected control group (n=51), 50% of the after-COVID controls (n=14), and 75.3% of the post-COVID Patients (n=52), showing a predominance of female participants in the post-COVID patient group; however, regression analysis showed that sex had no influence. Education level was measured using the International Standard Classification of Education (ISCED) scale, where the mean score for uninfected controls was 5.1 ± 0.45, slightly higher than both the after-COVID controls (4.95 ± 0.52) and post-COVID Patients (4.73 ± 0.23). Vaccination status was also recorded, revealing that vaccinated individuals were present in the three groups: uninfected controls (n=22), after-COVID controls (n=13), and post-COVID patients (n=44).

Regarding the characteristics of acute COVID-19 infection (**Supplementary Table S1**), among the after-COVID controls, 92.9% (n=26) had mild disease, while 7.1% (n=2) experienced severe disease requiring hospitalization. Similarly, 82.6% (n=57) reported a mild infection in the post-COVID patient group, and 17.4% (n=12) reported a severe illness. The mean time from acute infection to assessment was 10.6 ± 8.4 months for after-COVID controls and 15.1 ± 4.9 months for post-COVID patients, indicating that the latter group had a longer interval since infection, although this difference was not statistically significant.

As shown in **Supplementary Table S1**, patients with post-COVID exhibited a range of persistent symptoms. As expected, memory failure and poor concentration were the most reported manifestations, affecting 79.7% of post-COVID patients (n=55). Other common complaints included headaches (47.8%, n=33), fatigue/asthenia (37.7%, n=26), and myalgia/arthralgia (24.6%, n=17). Anxiety/depression affected 23.2% (n=16), while insomnia was noted by 18.8% (n=13). Additionally, symptoms such as dizziness or gait instability (14.5%, n=10), paresthesia (13%, n=9), respiratory alterations (10.1%, n=7), and smaller subsets of patients reported other persistent symptoms (18.8%, n=13).

Overall cognitive level score was assessed using the Addenbrooke’s Cognitive Examination (ACE III) test, with the post-COVID group showing varying degrees of cognitive impairment. Approximately 68.3% of post-COVID patients (n=41) had average score, z > -0.71, equivalent to the >24th percentile, which can be considered overall cognitively unimpaired using the ACEIII criteria. Around 20% of post-COVID patients (n=12) a low ACEIII average score, z ≤ -0.71, equivalent to the ≤24th percentile, indicating mild overall cognitive impairment. Finally, 11.7% of post-COVID patients (n=7) exhibited the lowest ACEIII average score, z ≤ -1.40, equivalent to the ≤8th percentile, revealing severe overall cognitive impairment.

### 3.2 Serum RAS autoantibodies. AT1-AA levels are significantly higher in post-COVID- Patients

In serum, the levels of the pro-inflammatory autoantibodies directed against AT1 receptors (AT1-AA) were significantly elevated in patients suffering from post-COVID compared to both uninfected individuals and those in the after-COVID control group (who had recovered from COVID-19 and did not exhibit neurological post-COVID symptoms). In the present cohorts, the values were for uninfected controls: 8.934 U/mL± 0.935 (mean± SEM), after-COVID controls: 11.588 U/mL ± 1.770 (mean± SEM), and post-COVID patients: 15.626 U/mL ± 1.273 (mean± SEM).

**Figure 2:**
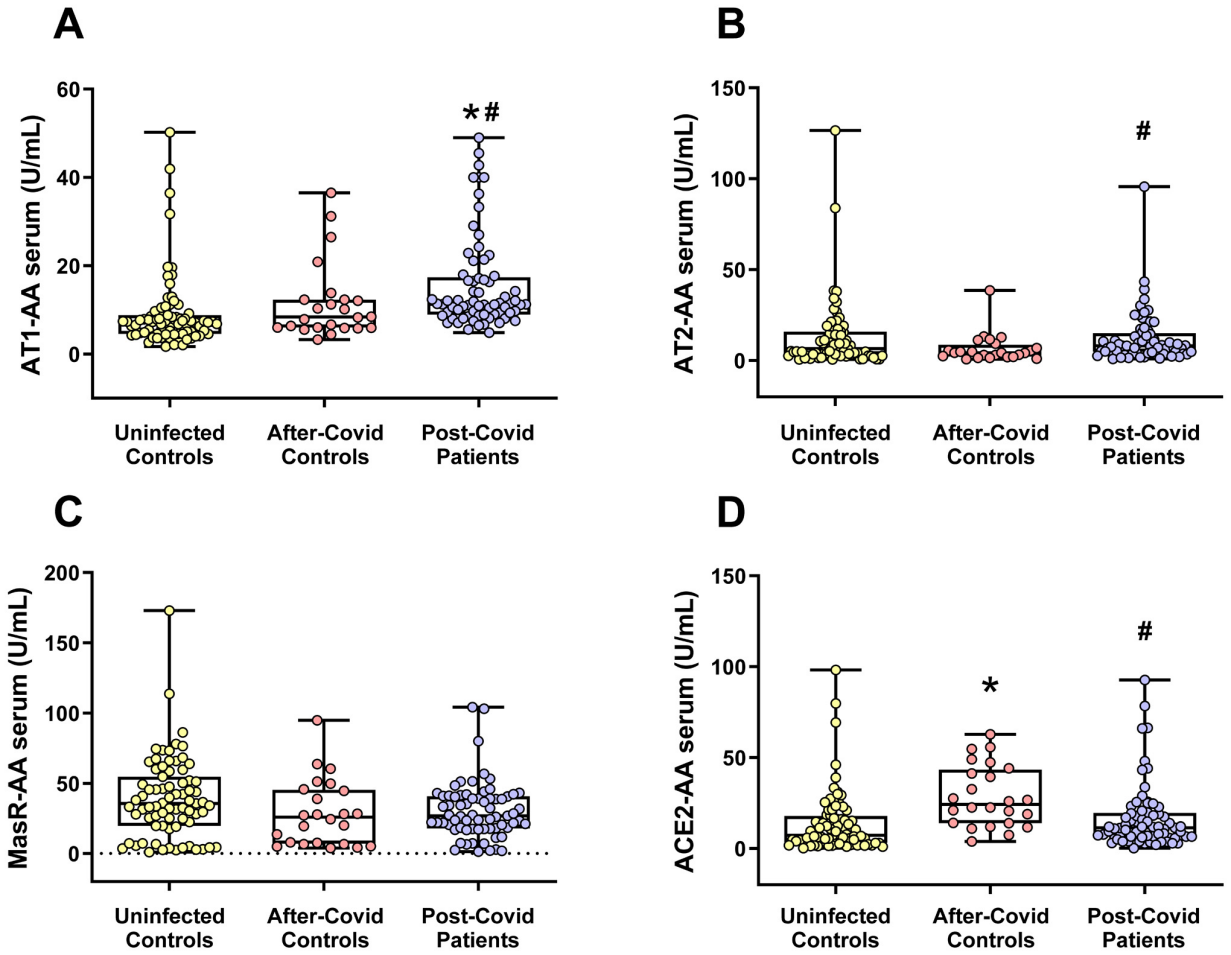
Serum levels of different RAS autoantibodies in the three studied groups. **(A)** AT1-AA serum levels were significantly higher in the post-COVID patient group. Changes in autoantibody levels for the RAS anti-inflammatory axis [i.e. AT2 **(B)** and MasR **(C)**] were less clear. **(D)** Serum levels of ACE2-AA were significantly higher in the after-COVID control group. Data distribution is shown using a box plot with boxes representing the IQR, i.e., the limits between the first quartile (Q1, 25%) and the third quartile (Q3, 75%). The whiskers extend up to maximum and minimum values. *p < 0.05 relative to uninfected control; ^#^ *p<*0.05 relative to after-COVID controls. Kruskal–Wallis One Way Analysis of Variance on Ranks with Dunn’s Method *post hoc* test. ACE2-AA, ACE2 Autoantibodies; AT1-AA, Autoantibodies for AT1 receptors; AT2-AA, Autoantibodies for AT2 receptors; IQR, Interquartile range; MasR-AA, Autoantibodies for Mas receptors.

Changes in levels of autoantibodies associated with the compensatory axis of the RAS (namely AT2-AA and MasR-AA) were less clear. Specifically, no significant alterations were observed in MasR-AA levels in post-COVID patients relative to other groups, and levels of AT2-AA were significantly higher in post-COVID patients than in the after-COVID control group, although this increase did not reach statistical significance when compared with uninfected patients. ACE2 autoantibody (ACE2-AA) levels were significantly decreased in the post-COVID patient group relative to the after-COVID control group, and similar to levels of uninfected controls (**Figures 2A–D**).

Vaccination status and age of patients are significantly associated with both AT1-AA and ACE2-AA. Multiple linear regression models were fitted with log-transformed autoantibody levels as the response variable, while patient group (after-COVID controls, uninfected controls, or post-COVID patients), age, and vaccination status were used as covariates. In both models, the reference category for the patient group was set as uninfected controls. The conclusions for both biomarkers were consistent with those obtained in univariate analyses, with AT1-AA levels being higher in post-COVID patients and ACE2-AA levels higher in after-COVID controls, both compared to uninfected controls. The model coefficients and significance levels are presented in **Supplementary Table S2** for AT1-AA and in **Supplementary Table S3** for ACE2-AA. When the reference category was instead set as after-COVID controls, the results mirrored those found in the univariate analysis: post-COVID patients significantly had higher levels of AT1-AA and lower levels of ACE2-AA than after-COVID controls.

### 3.3 NFL serum levels are significantly higher in post-COVID patients and correlate with AT1-AA serum levels

**Figure 3:**
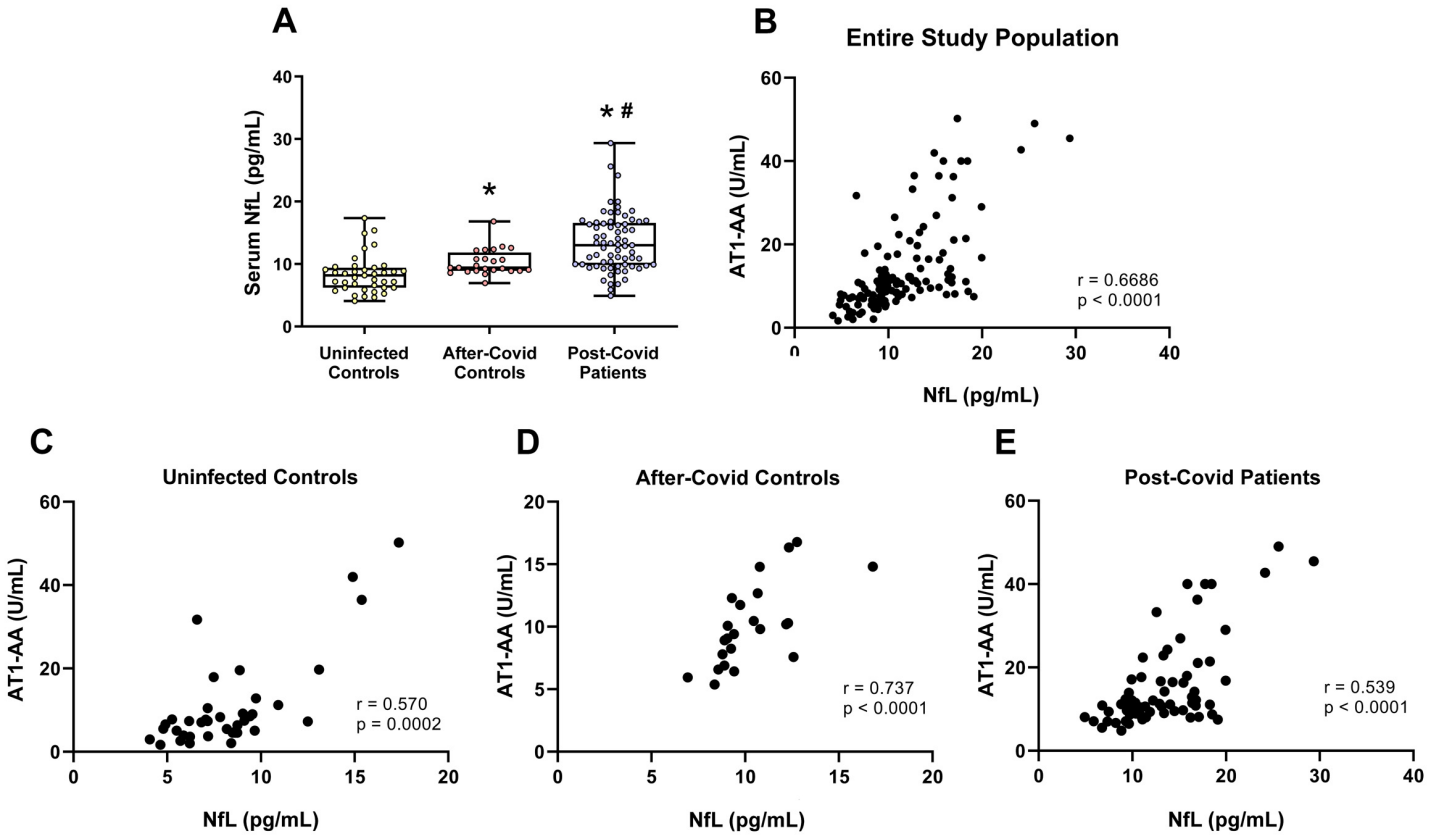
NfL serum levels in the three studied groups. **(A)** NfL serum levels were significantly higher in patients who had suffered COVID-19 but did not present post-COVID symptoms (after-COVID controls) and post-COVID patients than in uninfected controls and NfL levels in post-COVID patients were significantly higher than in after-COVID controls. Furthermore, levels of serum NfL significantly correlated with serum levels of AT1-AA in the entire study population **(B)**, uninfected controls **(C)**; after-COVID controls **(D)**, and post-COVID patients **(E)**. In **(A)**, data distribution is shown using a box plot with boxes representing the IQR, i.e., the limits between the first quartile (Q1, 25%) and the third quartile (Q3, 75%). The whiskers extend up to maximum and minimum values. *p < 0.05 relative to uninfected control; ^#^ *p<*0.05 relative to after-COVID controls. Kruskal–Wallis One Way Analysis of Variance on Ranks with Dunn’s Method *post hoc* test. In **(B–D)**, correlations between NfL serum levels and AT1-AA serum levels were assessed using Spearman’s rank correlation coefficient. AT1-AA, Autoantibodies for AT1 receptors; IQR, Interquartile range; NfL, Neurofilament light chain.

Serum NfL levels serve as a peripheral biomarker indicative of neuroaxonal damage. In this study, both after-COVID control and post-COVID patient groups displayed significantly higher serum NfL levels compared to uninfected individuals, with the post-COVID group exhibiting significantly higher NfL levels than those in the after-COVID control group (**Figure 3A**). Moreover, serum NfL levels were found to correlate significantly with AT1-AA levels across the entire study population (**Figure 3B**) and all patient groups (**Figures 3C–E**). However, these correlations were not found for the other autoantibodies (**Supplementary Figure S1**). A power analysis was conducted to verify whether the initially assumed large effect size was held. A large effect of the grouping variable was confirmed (f = 0.7), resulting in a power of 99% for this contrast. A positive correlation between age and NfL serum levels, as well as an increased presence of serum NfL levels in both after-COVID control and post-COVID patient groups compared to uninfected controls, remained significant in a multivariate model with potential confounders included as covariates (**Supplementary Table S4**). In addition, a recent study in the context of multiple sclerosis has suggested the correction of NfL values using a Z-score correction (35). After using the corresponding online calculator for age-adjusted and Body Mass Index (BMI)-adjusted values (https://shiny.dkfbasel.ch/baselnflreference/), the corrected NfL values in the after-COVID control group were also significantly higher than in the uninfected group, and values in the post-COVID patient group were significantly higher than in the after-COVID control and uninfected groups. Correlations with AT1-AA were also observed and, particularly, were present in the post-COVID patient group (**Supplementary Figure S2**).

### 3.4 CSF RAS autoantibodies: AT1-AA levels are significantly higher in post-COVID patients

**Figure 4:**
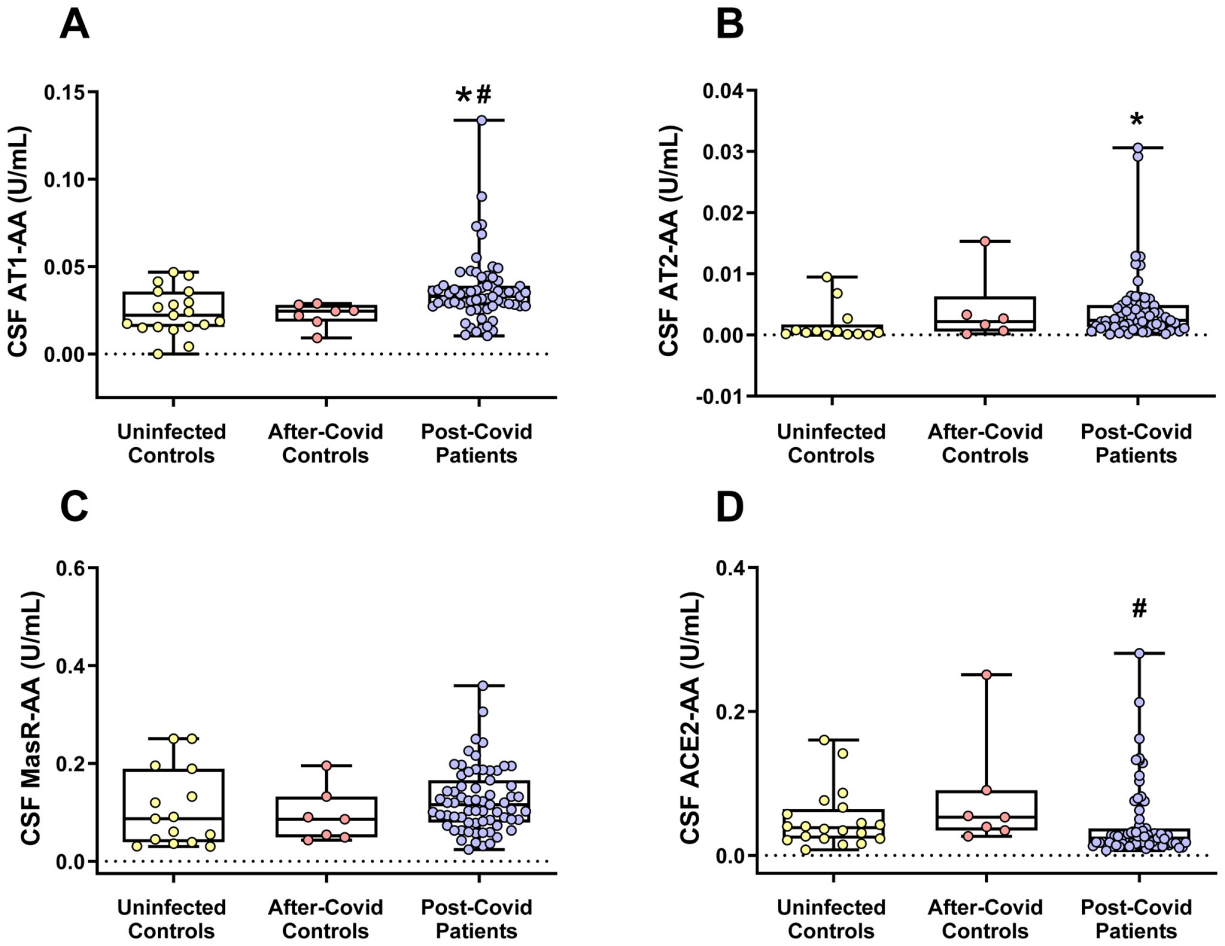
CSF levels of different RAS autoantibodies in the three studied groups. **(A)** CSF AT1-AA levels were significantly higher in the post-COVID patients. **(B)** CSF levels of AT2-AA were significantly higher in post-COVID patients than in uninfected patients but not significantly different compared to after-COVID controls. **(C)** In the post-COVID patient group, CSF MasR-AA levels exhibited a trend toward being higher than in the other groups, although this difference did not reach statistical significance. **(D)** CSF ACE2-AA levels were significantly lower in the post-COVID patient group than in the after-COVID control group. Data distribution is shown using a box plot with boxes representing the IQR, i.e., the limits between the first quartile (Q1, 25%) and the third quartile (Q3, 75%). The whiskers extend up to maximum and minimum values. *p < 0.05 relative to uninfected controls; ^#^ *p*<0.05 relative to after-COVID controls. Kruskal–Wallis One Way Analysis of Variance on Ranks with Dunn’s Method *post hoc* test. ACE2-AA, ACE2 Autoantibodies; AT1-AA, Autoantibodies for AT1 receptors; AT2-AA, Autoantibodies for AT2 receptors; IQR, Interquartile range; MasR-AA, Autoantibodies for Mas receptors.

In the cerebrospinal fluid (CSF) analysis (**Figure 4**), AT1-AA levels were significantly elevated in post-COVID patients compared to both uninfected individuals and after-COVID controls. In terms of the RAS compensatory axis, AT2-AA CSF levels were significantly higher in the post-COVID patient group compared to uninfected patients but did not differ significantly from those in the after-COVID control group. Furthermore, while CSF MasR-AA levels in post-COVID patients tended to be higher than in the other groups, this difference did not reach statistical significance. As observed in serum, CSF ACE2-AA levels were significantly lower in the post-COVID group compared to the after-COVID control group, which also points towards a higher compensatory response in the post-COVID patient group as ACE2-AA are antagonists of ACE2. There were no significant associations between AT1-AA, AT2-AA and ACE2-AA measured in CSF and the confounding factors considered. Consequently, no multivariate analysis was conducted at this stage.

### 3.5 CSF RAS autoantibodies post-COVID patients with different degrees of cognitive impairment

**Figure 5:**
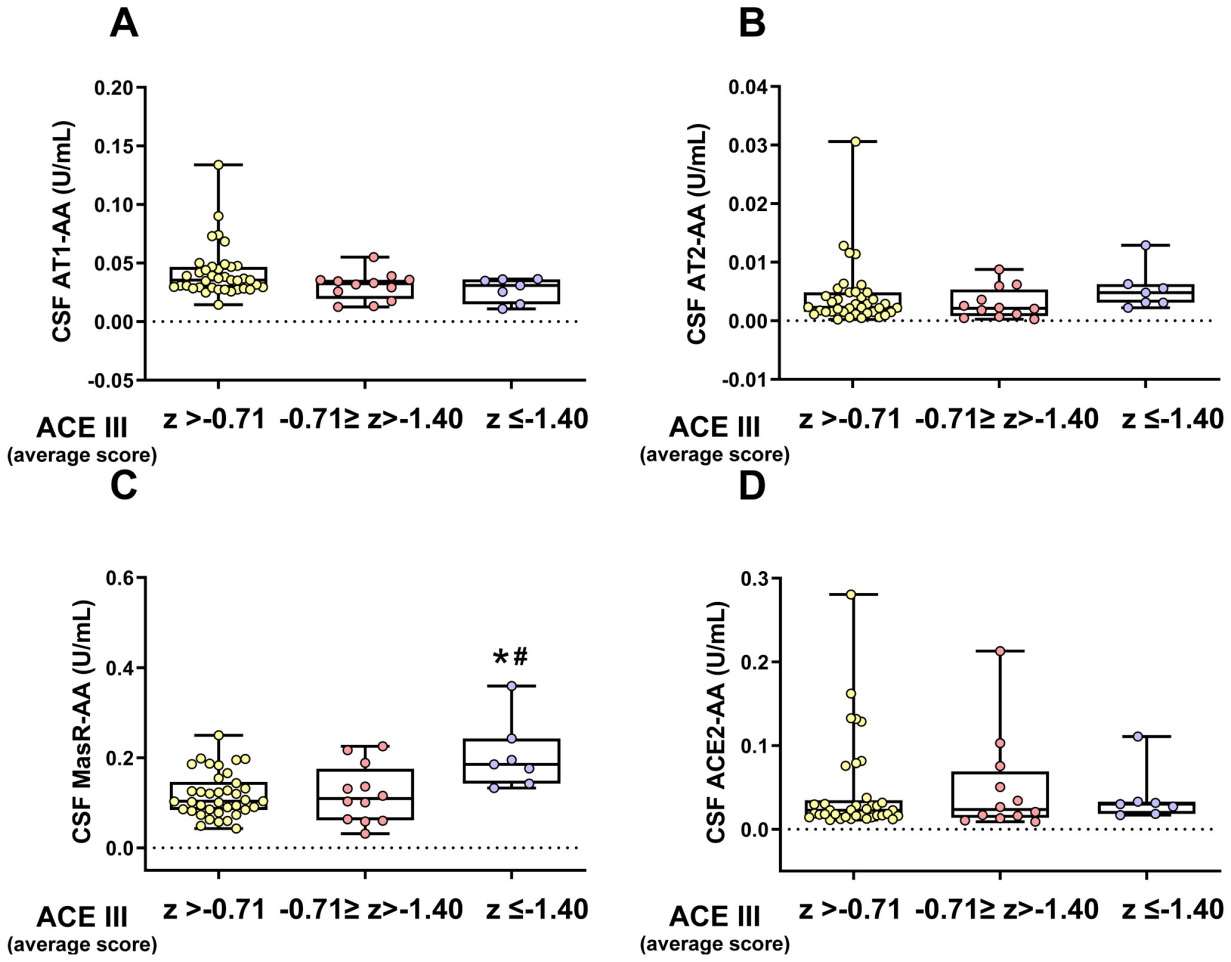
CSF levels of different RAS autoantibodies in post-COVID patients with different degrees of cognitive impairment. No significant differences in CSF AT1-AA or ACE2-AA levels were found between the three post-COVID subgroups: unimpaired overall CI (z > -0.71), mild overall CI (-0.71 ≥ z >-1.40), and severe overall CI (z ≤ -1.40), **(A, D)**. However, the group with more marked CI exhibited significantly higher CSF MasR-AA levels **(C)** and a trend toward higher CSF AT2-AA levels **(B)**, though the latter did not reach statistical significance. Data distribution is shown using a box plot with boxes representing the IQR, i.e., the limits between the first quartile (Q1, 25%) and the third quartile (Q3, 75%). The whiskers extend up to maximum and minimum values. *p < 0.05 relative to ACE III (Average score) of Z> -0.71; ^#^ *p<*0.05 relative to ACE III (Average score) of -071<Z>-1.40. Kruskal–Wallis One Way Analysis of Variance on Ranks with Dunn’s Method *post hoc* test. ACE2-AA, ACE2 Autoantibodies; AT1-AA, Autoantibodies for AT1 receptors; AT2-AA, Autoantibodies for AT2 receptors; CI, Cognitive Impairment; IQR, Interquartile range; MasR-AA, Autoantibodies for Mas receptors.

The post-COVID cohort with cognitive tests was subdivided into three groups based on the degree of overall cognitive impairment (CI) to further explore the potential relationship between RAS autoantibodies and cognitive impairment. Although CSF levels of the pro-inflammatory AT1-AA were significantly higher in post-COVID patients than in after-COVID and uninfected control groups (**Figure 4A**), no significant differences were observed in CSF levels of AT1-AA or ACE2-AA across these post-COVID subgroups. However, patients with the most pronounced cognitive impairment exhibited significantly higher levels of MasR-AA and a trend toward elevated AT2-AA levels, although the latter did not reach statistical significance (**Figure 5**). Since MasR-AA CSF showed an association with the vaccination status of patients, a multivariate analysis was conducted to determine whether the association with the patient group remained. Once again, a similar conclusion to that reached through univariate analyses was obtained (**Supplementary Table S5**).

### 3.6 Autoantibody correlations in post-COVID patients

**Figure 6:**
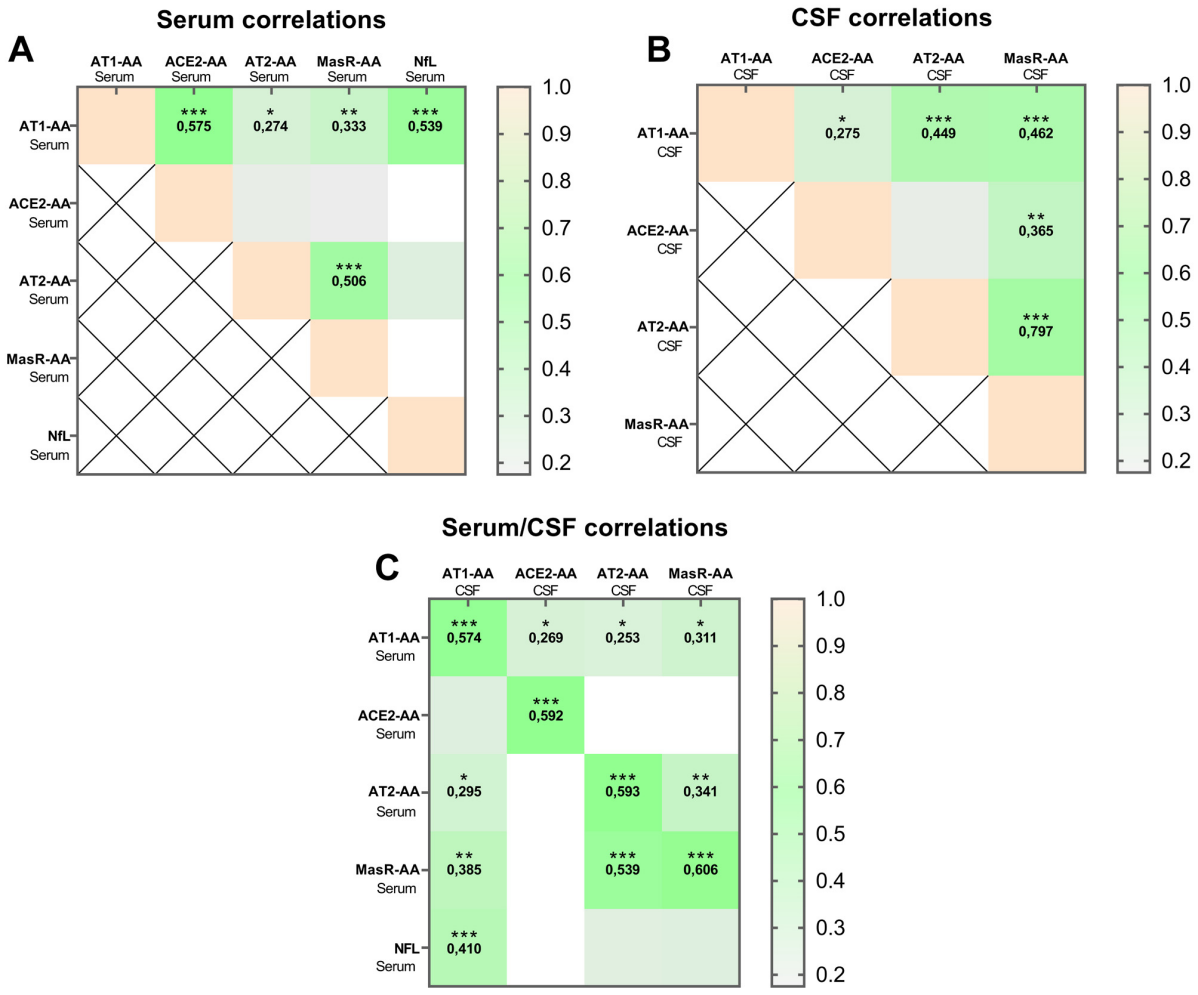
Autoantibody correlations in post-COVID patients. Results of Spearman’s correlation matrixes show variables (AT1-AA, ACE2-AA, AT2-AA, MasR-AA and NfL) correlating at varying levels of significance (*p < 0.05; **p < 0.01; ***p < 0.001) in serum **(A)**, CSF **(B)** and between serum and CSF **(C)**. AT1-AA, Autoantibodies for AT1 receptors; ACE2-AA, ACE2 Autoantibodies; AT2-AA, Autoantibodies for AT2 receptors; MasR-AA, Autoantibodies for Mas receptors; NfL, Neurofilament light chain.

Additionally, we investigated the correlations between various types of autoantibodies in both serum (**Figure 6A**) and CSF (**Figure 6B**), as well as across serum and CSF (**Figure 6C**). In serum, AT1-AA showed a significant correlation with ACE2-AA and NfL, while AT2-AA correlated with MasR-AA. In the CSF, significant correlations were observed between AT1-AA and both AT2-AA and MasR-AA, and a strong correlation was noted between AT2-AA and MasR-AA. Notably, moderate but significant correlations were also identified between serum and CSF levels of AT1-AA, AT2-AA, and MasR-AA. Additionally, CSF AT1-AA levels were significantly correlated with serum NfL levels (**Figure 6C**).

## 4 Discussion

Both in serum and CSF, the present study shows a significant increase in levels of AT1-AA in the post-COVID group of patients, which correlates with the levels of the neuroaxonal damage marker NfL. Recent studies from our laboratory and others showed a correlation between serum AT1-AA at diagnosis and severity outcome of COVID-19 (8, 9, 36). This association may be attributable to heightened activity of the RAS pro-inflammatory arm (i.e. AT1 overactivity), which drives pro-thrombotic, pro-fibrotic, and pro-inflammatory events, including elevated cytokine levels (17). In the COVID-19 acute period, disease-associated inflammation is likely to trigger the observed rise in AT1-AA levels, followed by some degree of the anti-inflammatory RAS compensatory response. These processes may persist in some patients, contributing to post-COVID development, as summarized in **Supplementary Figure S3**.

### 4.1 AT1-AA and neuroaxonal damage in post-COVID patients

Numerous previous studies have linked enhanced AT1 activation with the progression of neuroinflammation and neurodegeneration in various experimental models, including neurotrauma (37, 38), and Parkinson´s and Alzheimer´s disease (39–41). However, RAS is not just involved in enhancing the neuroinflammatory response (42), modulation of neurodegeneration (39, 40), and regulation of cognitive functions (43). A direct effect of RAS on neurons has been supported by human studies showing that dopaminergic neurons expressing high levels of the AT1 receptor gene are the most vulnerable to degeneration (44), as AT1 overactivity enhances NADPH-oxidase- and mitochondrial-derived oxidative stress and calcium raising (40). Other studies have observed that variants of the gene that encodes AT1 receptor were associated with accelerated hippocampal volume loss over time (45), and elevated Ang II levels diminished total grey matter volume and reduced volumes in regions crucial for memory and executive functions, such as the hippocampus (46). In addition, several studies have shown that activation of endothelial AT1 receptors plays a critical role in BBB disruption, and that hypertension-induced BBB permeability is blocked by AT1 antagonists and not by other antihypertensive drugs, which suggests that activation of endothelial AT1 receptors is responsible for the increase in BBB permeability and not the hypertension itself (47–49). The decrease in ACE2 activity and the subsequent decrease in levels of Ang 1–7 at the BBB may also contribute to the increase in BBB permeability, which may be promoted by the antagonistic effect of ACE2-AA (50).

The generation of AT1-AA has been related to the release of proinflammatory cytokines during inflammatory processes, as infusion of IL-6 and TNF-α in animal models induced an increase in levels of AT1-AA (25, 51). Upregulation of the cytokine TNFSF14 (TNF superfamily member 14, LIGHT), acting via tissue transglutaminase 2 (TG2), appears to play a key role (52). TG2-mediated alterations of AT1 receptors (53, 54) may result in the formation of neoantigens that promote AT1-AA generation (53). Our previous research corroborated the significant correlation between AT1-AA and LIGHT levels in COVID-19 patients (9) and in animal models and patients with neurodegenerative diseases (12, 28, 30).

The binding site of these autoantibodies to AT1R was first demonstrated by Wallukat et al. a couple of decades ago, showing that the autoantibodies are directed at the second extracellular loop of the AT1 receptor and activate the receptor similarly to its natural agonist Ang II (23). AT1-AA are potent activators of the RAS pro-oxidative pro-inflammatory axis, not only because they act as agonists of the main RAS pro-inflammatory receptor (AT1), but because the binding of AT1-AA stabilizes AT1 receptors in a permanent activation by blocking AT1 internalization (55), thus upregulating the AT1 receptor expression and AT1 receptor sensitization (52, 54, 56). AT1R internalization after Ang II binding is a major mechanism for mitigating sustained receptor activation (57, 58), which is blocked by AT1-AA binding. Consistent with this overactivation of AT1 receptors by AT1-AA in neurons and glial cells, we have recently shown that, in cell cultures, AT1-AA activate major pathways stimulated by AT1 activation, inducing intraneuronal calcium increase and NADPH-oxidase activation (29, 30, 59), exacerbating neuronal degeneration, and increasing levels of pro-inflammatory cytokines in the culture medium (28, 30). In rat models, systemic administration of AT1-AA via minipumps disrupted the BBB, exacerbated neuronal degeneration, and increased brain levels of neuroinflammation markers (28, 30). In our experimental models, the effects of AT1-AA were blocked *in vivo* and *in vitro* by AT1 receptor blockers such as candesartan and telmisartan, confirming that the effects are via AT1. In addition to other possible factors, such as a concomitant increase in proinflammatory cytokines due to the inflammatory process, the presence of high levels of circulating AT1-AA may contribute to the disruption of the BBB (see above), leading to its permeability to autoantibodies. However, several recent studies also suggest the passage of activated B cells (activated by neoantigens from the corresponding RAS receptor in this case) through the BBB and the intrathecal formation of autoantibodies (60–62). Interestingly, recent data suggest a requirement for naive antigen-specific B cell recruitment into the dural-associated lymphoid tissues (DALT) from the circulation, but once they have been locally activated, a germinal center response is held in the DALT independently of circulating immune cell input (60). Our data on AT1-AA and ACE2-AA in Parkinson’s patients using the corrected antibody index point to the intrathecal formation of CSF RAS autoantibodies (28).

In the present study, the increase in serum levels of a marker of neuroaxonal damage (Nfl) correlated with levels of AT1-AA. The findings of a cross-sectional study are correlative rather than causal; however, the present results, together with the above-mentioned experimental data showing AT1-AA-induced neuronal damage, suggest a role of the increased AT1-AA in the neuroaxonal damage in post-COVID patients. Conversely, neuroinflammatory and neuroaxonal damage can promote a further increase in autoantibody generation through the above-mentioned mechanisms. Altogether, this suggests the involvement of AT1-AA in brain lesions and neurological symptoms observed in post-COVID patients in the present and other recent studies (3, 6). The present results are consistent with a recent study observing that post-COVID patients with cognitive impairment showed significantly higher NfL levels than healthy controls and post-COVID patients without these symptoms (63).

### 4.2 RAS compensatory axis AA and neuroaxonal damage in post-COVID patients

Inflammatory responses often provoke compensatory anti-inflammatory mechanisms aimed at curbing excessive immune activation (64, 65). Consistent with this, a recent study reported the presence of autoantibodies targeting pro-inflammatory chemokines, which was associated with favorable COVID-19 outcomes and inversely correlated with post-COVID development (66). To study possible compensatory mechanisms in the RAS context, we explored agonistic autoantibodies targeting the RAS anti-inflammatory axis (AT2 and Mas receptors). These autoantibodies could mitigate the effects of the persistent AT1-AA elevation observed in post-COVID patients. Upregulation of compensatory RAS components such as AT2 and MasR is known to counteract AT1 overactivity (58, 67, 68), as AT2 and MasR activation promotes release of anti-inflammatory cytokines such as IL-10 and downregulates levels of pro-inflammatory cytokines such as IL-6 and others (69, 70). Furthermore, increased expression of AT2 and Mas receptors in damaged cells may lead to increased release of the corresponding antigens, leading to an increase in agonistic AT2 and MasR autoantibodies. However, no consistent increase in AT2-AA or MasR-AA levels was detected in serum to offset the peripheral effects of pro-inflammatory AT1-AA in post-COVID patients. AT2-AA levels were significantly higher in post-COVID patients than in after-COVID control group, and not significantly higher than in uninfected patients. A more consistent compensatory response was observed in the CSF, with significantly elevated AT2-AA levels, relative to controls, and a trend towards increased MasR-AA levels, although the latter did not reach statistical significance. Consistent with this, significant correlations were found between CSF AT1-AA, CSF AT2-AA, and CSF MasR-AA levels, with a strong correlation between CSF AT2-AA and MasR-AA.

ACE2-AA play an antagonist effect on ACE2 activity (19, 22), downregulating the RAS anti-inflammatory arm and promoting the pro-inflammatory RAS effects. Consistent with this, an increase in ACE2-AA was observed in severe COVID-19 patients in previous studies from our laboratory and several others (9, 35). In the present study, we observed significantly lower levels of ACE2-AA in post-COVID patients relative to the after-COVID controls, which also points to the upregulation of RAS compensatory responses in post-COVID patients, as ACE2-AA are antagonists of ACE2.

### 4.3 RAS autoantibodies and degree of cognitive impairment

As discussed above, dysregulation of brain RAS by AA may contribute to brain lesions and neurodegenerative processes, including regions responsible for cognitive function (43, 71, 72). To further explore the possible role of RAS autoantibodies in cognitive impairment, we stratified post-COVID patients into three subgroups based on the severity of their ACEIII scores for overall cognitive impairment and analyzed the levels of RAS AA in the CSF. Although all subgroups showed higher AT1-AA levels than non-post-COVID groups, no significant difference in AT1-AA levels was detected between the three post-COVID subgroups. Unexpectedly, we observed significantly higher levels of MasR-AA and a trend to higher levels of AT2-AA in the CSF of patients with the most marked cognitive impairment, which suggests a stronger compensatory response in those patients with more COVID-19-related neuropathological damage, leading to more severe cognitive impairment (73).

In addition, recent studies have suggested a major role of hippocampal neurogenesis in the adult brain in cognitive processes and their dysregulation. Interestingly, several experimental studies have shown that RAS dysregulation may affect compensatory adult neurogenesis and neuroplasticity (74, 75). A direct regulation of neurogenesis by activation of RAS receptors located in neurogenic niches has been shown in our laboratory and others using animal and *in vitro* models (74–76). Activation of the AngII/AT1 axis inhibits neurogenesis, and activation of the ACE2/AT2/MasR axis promotes neurogenesis (74–76). Therefore, the overactivation of hippocampal AT1 receptors by an increase in AT1-AA observed in post-COVID patients may also promote the impairment of adult neurogenesis, contributing to cognitive deficits. The AT2/MasR compensatory response may be insufficient to counteract the inhibitory effects of AT1 overactivity in the post-COVID group of patients.

### 4.4 Limitations and conclusions

The results show the interest of measuring blood NfLs and AT1-AA in post-COVID patients with neurological symptoms such as cognitive impairment, despite the difficulty of performing accurate studies with clinical tests. A recurring source of discrepancies within and between studies on post-COVID is that biomarker-based results do not stratify/correlate with the clinical score. The lack of reliable clinical scoring and universal clinical tests yield different results, and this is further complicated by the heterogeneity of post-COVID etiopathogenesis and the diversity of the recruited cohorts in different studies. Post-COVID is a highly heterogeneous entity and may result from various underlying mechanisms. The present study includes a cohort of post-COVID patients with neurological symptoms, which may differ from other cohorts that may include a small number of patients with this clinical profile, which may lead to different results on NfLs and CSF RAS autoantibodies, particularly AT1-AA. Furthermore, we cannot rule out that some individuals from the uninfected control group may have had an asymptomatic infection, although this would lead to a reduction in differences with the post-COVID patient group rather than the opposite. In the present study, the results from subgroups of patients with different CI should be taken with caution due to the low number of patients analyzed, particularly in the most affected subgroup. The cross-sectional design, typical in early-stage research, doesn’t establish causality, but it lays a solid foundation for longitudinal studies and is supported by previous functional studies. Although the study has certain limitations, they do not significantly detract from the overall findings. The relatively small sample size, while limited, provides valuable insights into the trends observed, and similar results in future larger studies could further confirm these findings. While additional biomarkers could be explored, the focus on RAS-AA and NfL was appropriate for this study’s scope.

In conclusion, post-COVID is a highly heterogeneous entity, and different factors and mechanisms, including several types of AA, may be involved in different post-COVID symptoms and “phenotypes” (18, 77). In the present cohort of neurological post-COVID patients, the results suggest that persistent brain RAS dysregulation, particularly the persistent elevation of AT1-AA, which correlated with neuroaxonal damage markers and cognitive impairment, may play a significant role in the neurological symptoms associated with post-COVID. This is consistent with previous experimental data. Furthermore, the use of NfL and AT1-AA as biomarkers could aid in the early identification of CNS involvement in patients with a history of previous SARS-CoV-2 infection with neurological symptoms as those observed in the present study. Possible neurological post-COVID patients could be tested for levels of serum AT1-AA and NfL, as high levels of these markers help to support the CNS involvement rather than other possible origins of the claimed symptoms. However, additional confirmatory studies in larger and different populations are necessary. Furthermore, the present findings suggest the potential of targeting AT1 receptors as a therapeutic strategy for mitigating cognitive deficits in neurological post-COVID patients, particularly in subgroups of patients showing upregulated AT1-AA levels. Future research should focus on the possible therapeutic use of AT1 receptor blockers, which have shown promise in preclinical models for improving neurogenesis and cognitive function.

## Data Availability

The original contributions presented in the study are included in the article/**Supplementary Material**. Further inquiries can be directed to the corresponding authors.
