# Supplementary material for: Serum angiotensin type-1 receptor autoantibodies and neurofilament light chain as markers of neuroaxonal damage in post-COVID patients
Source: Front Immunol. 2025 Apr 22;16:1571027. doi: 10.3389/fimmu.2025.1571027 (PMC12052551; doi:10.3389/fimmu.2025.1571027)
Supplement: Supplementary file 1 [file DataSheet1.pdf]

Supplementary Material

1 Supplementary Figures and Tables

	Uninfected Controls N=99	After-COVID Controls N=28	Post-COVID Patients N=69	P[1]
**Demographics**				
Age (years, mean± SEM)	61.3±9.2	53.4±14	52.5±8.1	0.389
Sex (female) n (%)	51 (51.5)	14 (50)	52 (75.3)	0.004** U*vs*AC and U*vs*PC
Education level Scale (ISCED)[2] (mean± SEM)	5.1±0.45	4.95±0.52	4.73±0.23	0.340
Vaccinated n	22	13	44	[3]*(U*vs*AC) and ** (U*vs*PC)
**Acute COVID 19 infection**				
Mild disease n (%)	n/a	26 (92.9)	57 (82.6)	0.326
Severe disease (Hospitalisation) n (%)	n/a	2 (7.1)	12 (17.4)	0.326
Months from acute infection (mean± SEM)	n/a	10.6±8.4	15.1±4.9	0.992
**Post-COVID symptoms**				
Memory failure n (%)	n/a	n/a	55 (79.7)	n/a
Poor concentration n (%)	n/a	n/a	55 (79.7)	n/a
Headache n (%)	n/a	n/a	33 (47.8)	n/a
Fatigue/Asthenia n (%)	n/a	n/a	26 (37.7)	n/a
Myalgia/Arthralgia n (%)	n/a	n/a	17 (24.6)	n/a
Anxiety/Depression n (%)	n/a	n/a	16 (23.2)	n/a
Insomnia n (%)	n/a	n/a	13 (18.8)	n/a
Dizziness/Gait instability n (%)	n/a	n/a	10 (14.5)	n/a
Paresthesias n (%)	n/a	n/a	9 (13)	n/a
Respiratory alterations n (%)	n/a	n/a	7 (10.1)	n/a
Other persistent symptoms[4] n (%)	n/a	n/a	13 (18.8)	n/a
**Overall cognitive level (ACEIII test)**				
Unimpaired n (%)	n/a	n/a	41 (68.3)	n/a
Mildly impaired n (%)	n/a	n/a	12 (20)	n/a
Severe impaired n (%)	n/a	n/a	7 (11.7)	n/a

[1]P values refer to ANOVA, Kruskal-Wallis test or two-independent sample t-test for continuous variables or Chi-square for categorical variables, as appropriate (AC: After-COVID controls; PC: Post-COVID patients; U: Uninfected Controls). P-value *p≤0.05, **p≤0.01

[2] The International Standard Classification of Education (ISCED) is the official framework used to facilitate international comparisons of education systems). Scale from 0 (Early childhood education) to 8 (Doctoral degree or equivalent tertiary education level)

[3]Pairwise Chi-squared tests with Bonferroni correction were conducted to assess pairwise differences between vaccination status in the different groups. Statistically significant differences were found between Uninfected Controls and After-Covid Controls (p=0.034), and between Uninfected Controls and Post-Covid patients (p<0.001). In contrast, the difference between After-Covid controls and Post-Covid patients did not reach statistical significance (p=0.348).

[4] Other persistent symptoms include: Anosmia, Ageusia, temperature sensitivity, vision alterations, audition alterations and tinnitus, gastrointestinal problems, skin diseases and hair loss

SUPPLEMENTARY TABLE 2. Results of the multiple linear regression model using the logarithm of AT1-AA serum levels as the response variable.

Covariate	Category	Reference	Coefficient	SE	Z value	P-value
(Intercept)			1.969	0.077	25.415	<0.001
Condition	After-Covid Controls	Uninfected controls	-0.006	0.172	-0.034	n.s.
Condition	Post-COVID patients	Uninfected controls	0.348	0.125	2.794	<0.01
Vaccination status	Vaccinated	Non-vaccinated	0.203	0.125	1.624	n.s.
Age			-0.139	0.054	-2.588	<0.05

The model includes patient group (After-COVID controls, uninfected controls, or Post-COVID patients), age, and vaccination status as covariates. Coefficients (β), standard errors (SE), contrast statistics (Z) and p-values are reported. n.s., no significant.

SUPPLEMENTARY TABLE 3. Results of the multiple linear regression model using the logarithm of ACE2-AA serum levels as the response variable.

Covariate	Category	Reference	Coefficient	SE	Z value	P-value
(Intercept)			1.923	0.141	13.594	<0.001
Condition	After-Covid Controls	Uninfected controls	0.652	0.316	2.065	<0.05
Condition	Post-COVID patients	Uninfected controls	-0.098	0.229	-0.427	n.s.

Vaccination status	Vaccinated	Non-vaccinated	0.576	0.229	2.511	<0.05
Age			-0.031	0.098	-0.316	n.s.

The model includes patient group (After-COVID controls, uninfected controls, or Post-COVID patients), age, and vaccination status as covariates. Coefficients (β), standard errors (SE), contrast statistics (Z) and p-values are reported. n.s., no significant.

SUPPLEMENTARY TABLE 4. Results of the multiple linear regression model using the logarithm of NfL serum levels as the response variable.

Covariate	Category	Reference	Coefficient	SE	Z value	P-value
(Intercept)			1.415	0.104	13.588	<0.001
Condition	After-Covid Controls	Uninfected controls	0.269	0.078	3.462	<0.001
Condition	Post-COVID patients	Uninfected controls	0.328	0.060	5.441	<0.001
Vaccination status	Vaccinated	Non-vaccinated	-0.141	0.060	-2.360	<0.05
Age			0.027	0.027	0.980	n.s.
Sex	Woman	Man	0.039	0.053	0.731	n.s.
AT1-AA Serum levels			0.342	0.040	8.494	<0.001

The model includes patient group (After-COVID controls, uninfected controls, or Post-COVID patients), age, sex, vaccination status and AT1-AA serum levels as covariates. Coefficients (β), standard errors (SE), contrast statistics (Z) and p-values are reported. n.s., no significant.

SUPPLEMENTARY TABLE 5. Results of the multiple linear regression model using the logarithm of MasR-AA serum levels as the response variable.

Covariate	Category	Reference	Coefficient	SD	Z value	P-value
(Intercept)			-2.005	0.168	-11.909	<0.001
Impaired	$-0.71 \geq Z > -1.40$	$Z > -0.71$	-0.210	0.146	-1.438	n.s.
Impaired	$Z \leq -1.40$	$Z > -0.71$	0.491	0.232	2.121	<0.05
Vaccination status	Vaccinated	Non-Vaccinated	-0.197	0.154	-1.284	n.s.

The model includes the impairment level and the vaccination status of patients. Coefficients (β), standard errors (SE), contrast statistics (Z) and p-values are reported. n.s., no significant.

1.1 Supplementary Figures

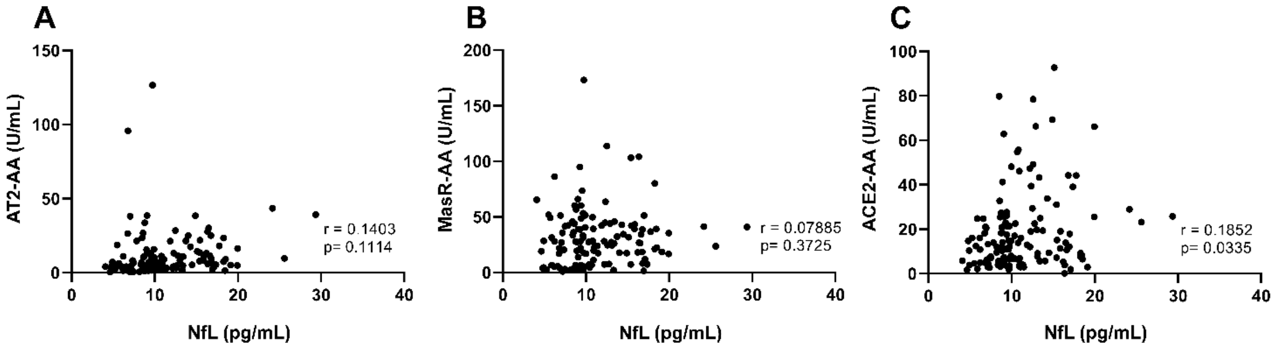

Supplementary Figure 1. Serum Correlations Between NfL and Autoantibodies in the entire Study Population. Serum NfL levels showed no strong correlation with AT2-AA **(A)**, MasR-AA **(B),** or ACE2-AA **(C)** levels. However, the association between NfL and ACE2-AA was statistically significant. Correlations were evaluated using Spearman's rank correlation coefficient. ACE2-AA, autoantibodies against ACE2; AT2-AA, autoantibodies against AT2 receptors; MasR-AA, autoantibodies against MasR receptors; NfL, neurofilament light chain.

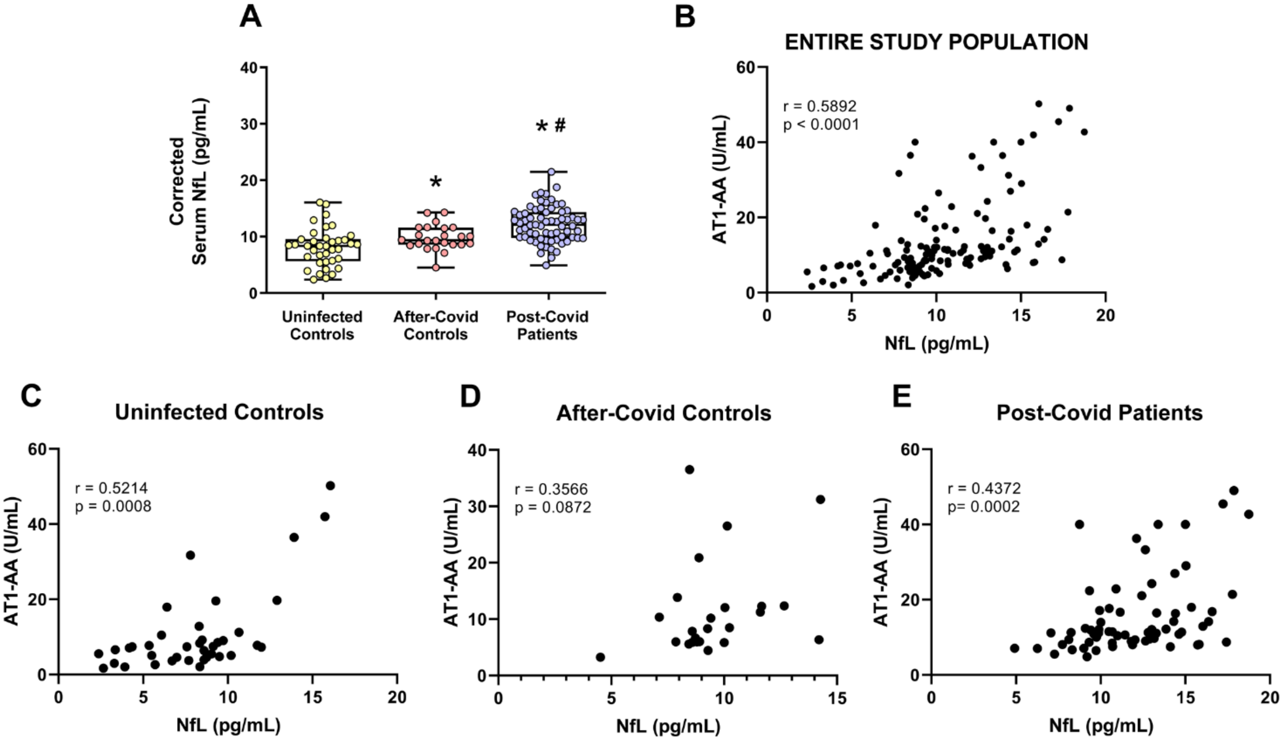

Supplementary Figure 2. Serum NfL levels corrected for age and BMI in COVID-19 patients and uninfected controls, calculated using the published Z-score method (35) and the online tool: https://shiny.dkfbasel.ch/baselnflreference/. **(A).** Serum NfL levels were significantly higher in both After-COVID controls and Post-COVID patients compared to uninfected controls. Moreover, NfL levels were significantly higher in Post-COVID patients than in After-COVID controls. Furthermore, serum levels of NfL significantly correlated with serum levels of AT1-AA in the entire study population **(B),** uninfected controls **(C),** and post-COVID patients **(E)**. In **A**, data are presented as box plots: boxes represent the interquartile range (IQR), i.e., the range from the first quartile (Q1, 25%) to the third quartile (Q3, 75%), and whiskers indicate the minimum and maximum values. $^*p < 0.05$ vs. uninfected controls; $p < 0.05$ vs. post-COVID controls. Statistical analysis was performed using one-way ANOVA followed by the Student–Newman–Keuls post hoc test. In **B–E**, correlations were assessed using Spearman's rank correlation coefficient. AT1-AA: Autoantibodies for AT1 receptors; BMI: Body Mass Index; IQR: Interquartile range; NfL: Neurofilament light chain.

35. Benkert P, Meier S, Schaedelin S, Manouchehrinia A, Yaldizli O, Maceski A, et al. Serum Neurofilament Light Chain for Individual Prognostication of Disease Activity in People with Multiple Sclerosis: A Retrospective Modelling and Validation Study. *Lancet Neurol.* (2022) 21:246-57 doi: 10.1016/S1474-4422(22)00009-6.

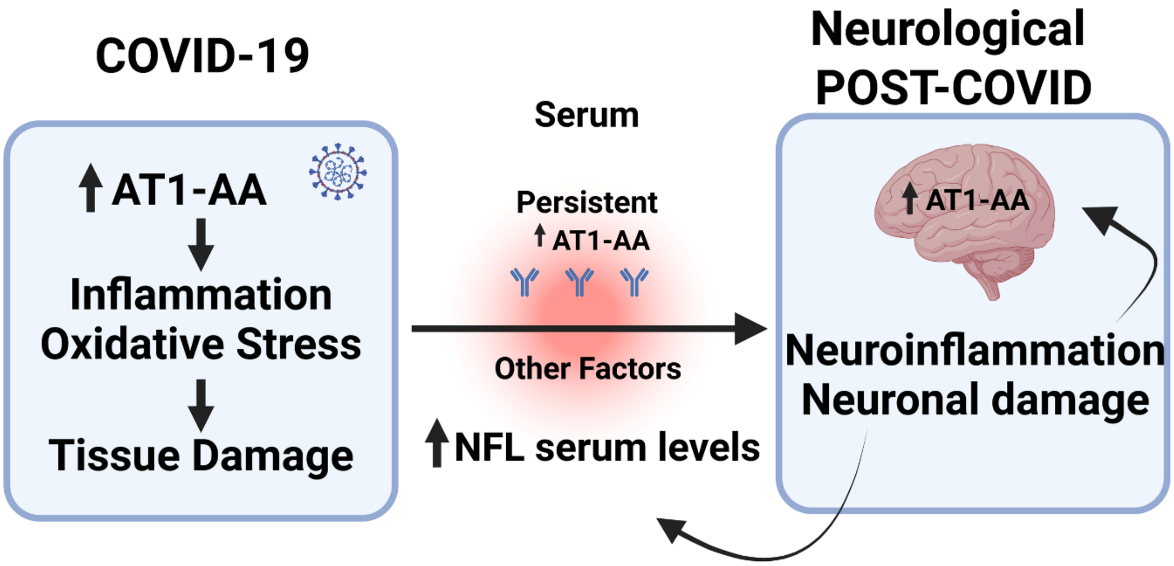

Supplementary Figure 3. Proposed mechanism linking persistent AT1-AA to neuroinflammation and neuronal damage in post-COVID condition. Schematic representation of the hypothesized pathogenic pathway. During acute COVID-19, increased levels of angiotensin II type 1 receptor autoantibodies (AT1-AA) promote inflammation, oxidative stress, and tissue damage. In a subset of patients, AT1-AA persist in the serum during the post-COVID period, potentially contributing, along with other unknown factors, to neuroinflammation and neuroaxonal injury as indicated by elevated neurofilament light chain (NFL) serum levels. Figure created using Biorender (https://biorender.com/).
